# Quantifying the magnitude of the general contextual effect in a multilevel study of SARS-CoV-2 infection in Ontario, Canada: application of the median rate ratio in population health research

**DOI:** 10.1186/s12963-024-00348-8

**Published:** 2024-10-07

**Authors:** Tristan Watson, Jeffrey C. Kwong, Kathy Kornas, Sharmistha Mishra, Laura C. Rosella

**Affiliations:** 1 Dalla Lana School of Public Health, Health Sciences Building 6th floor, 155 College Street, Toronto, ON M5T 3M7 Canada; 2 ICES, G1 06 2075 Bayview Ave, Toronto, ON M4N 3M5 Canada; 3 Public Health Ontario, 661 University Ave Suite 1701, Toronto, ON M5G 1M1 Canada; 4 Department of Family and Community Medicine, Faculty of Medicine, University of Toronto, 6 Queen’s Park Crescent West 3rd Floor, Toronto, ON M5S 3H2 Canada; 5 University Health Network, 200 Elizabeth St, Toronto, ON M5G 2C4 Canada; 6 Li Ka Shing Knowledge Institute, St. Michael’s Hospital, Unity Health Toronto, 209 Victoria St, Toronto, ON M5B 1T8 Canada; 7 Division of Infectious Diseases, Department of Medicine, University of Toronto, 6 Queen’s Park Crescent West 3rd Floor, Toronto, ON M5S 3H2 Canada; 8 Institute for Better Health, Trillium Health Partners, 100 Queensway West, Mississauga, ON L5B 1B8 Canada; 9 Department of Laboratory Medicine and Pathology, Temerty Faculty of Medicine, University of Toronto, 1 King’s College Cir, Toronto, ON M5S 1A8 Canada; 10 Centre for Vaccine Preventable Diseases, University of Toronto, Toronto, Canada

**Keywords:** COVID-19, SARS-CoV-2, Multilevel analysis, Social determinants of health, General contextual effect, Median rate ratio, Poisson regression, Heterogeneity

## Abstract

**Background:**

Regional variations in SARS-CoV-2 infection were observed in Canada and other countries. Studies have used multilevel analyses to examine how a context, such as a neighbourhood, can affect the SARS-CoV-2 infection rates of the people within it. However, few multilevel studies have quantified the magnitude of the general contextual effect (GCE) in SARS-CoV-2 infection rates and assessed how it may be associated with individual- and area-level characteristics. To address this gap, we will illustrate the application of the median rate ratio (MRR) in a multilevel Poisson analysis for quantifying the GCE in SARS-CoV-2 infection rates in Ontario, Canada.

**Methods:**

We conducted a population-based, two-level multilevel observational study where individuals were nested into regions (i.e., forward sortation areas [FSAs]). The study population included community-dwelling adults in Ontario, Canada, between March 1, 2020, and May 1, 2021. The model included seven individual-level variables (age, sex, asthma, diabetes, hypertension, congestive heart failure, and chronic obstructive pulmonary disease) and four FSA census-based variables (household size, household income, employment, and driving to work). The MRR is a median value of the rate ratios comparing two patients with identical characteristics randomly selected from two different regions ordered by rate. We examined the attenuation of the MRR after including individual-level and FSA census-based variables to assess their role in explaining the variation in rates between regions.

**Results:**

Of the 11 789 128 Ontario adult community-dwelling residents, 343 787 had at least one SARS-CoV-2 infection during the study period. After adjusting for individual-level and FSA census-based variables, the MRR was attenuated to 1.67 (39% reduction from unadjusted MRR). The strongest FSA census-based associations were household size (RR = 1.88, 95% CI: 1.71–1.97) and driving to work (RR = 0.68, 95% CI: 0.65–0.71).

**Conclusions:**

The individual- and area-level characteristics in our study accounted for approximately 40% of the between-region variation in SARS-CoV-2 infection rates measured by MRR in Ontario, Canada. These findings suggest that population-based policies to address social determinants of health that attenuate the MRR may reduce the observed between-region heterogeneity in SARS-CoV-2 infection rates.

**Supplementary Information:**

The online version contains supplementary material available at 10.1186/s12963-024-00348-8.

## Background

The coronavirus disease 2019 (COVID-19) pandemic in Canada and other countries was marked by regional variations in severe acute respiratory syndrome coronavirus 2 (SARS-CoV-2) infection [1–3]. An extensive evidence base has reported socioeconomic inequalities over time in SARS-CoV-2 infection [4]. Socioeconomic inequalities in SARS-CoV-2 infection have also been reported between regions; that is, regions with the highest infection rates often coincided with a high proportion of socially disadvantaged population groups [5–9]. The extent to which the heterogeneity of SARS-CoV-2 infection between regions is associated with individual-level factors (e.g., age, sex, income, employment) and area-level factors (e.g., population density) is challenging to parse. For instance, a systematic review focused on socioeconomic inequalities in SARS-CoV-2 infection reported that people living in areas with high population density were more likely to come into close contact with others and have a higher risk of SARS-CoV-2 infection [10]. Measuring how individual- and area-level factors may explain heterogeneity in SARS-CoV-2 infection requires relevant multilevel data and the application of multilevel methods while considering potential sources of biases in the multilevel study design [11–13].

Key features of multilevel analyses are the ability to model the relationship, measure heterogeneity, and partition variance between individual-level factors and area-level factors on individual outcomes [14, 15]. These methods are conceptually consistent with our current understanding that the risk of SARS-CoV-2 infection is influenced by the socioecological contexts (e.g., home, workplace, neighbourhood) in which people live. In the multilevel analysis literature, the general contextual effect (GCE) describes how an individual’s context influences the individual outcomes [16]. Studies have used multilevel analyses to examine how a context, such as a neighbourhood, can affect the SARS-CoV-2 infection rates of the people within it [17–19]. However, few multilevel studies have quantified the magnitude of the GCE in SARS-CoV-2 infection rates and assessed how it may be associated with individual- and area-level characteristics [20, 21].

The median rate ratio (MRR) is a summary measure that quantifies the magnitude of GCE in multilevel Poisson regression [22]. There are also similar measures for different multilevel analyses. The median odds ratio (MOR) is used in multilevel logistic regression [22], and the median hazard ratio is used in multilevel survival analyses [23]. The MRR is the median value of the rate ratio comparing two patients with identical measured characteristics randomly selected from two different areas, where the higher rate is the numerator, and the lower rate is the denominator. If we were to compute the rate ratios across all possible randomly selected pairs of individuals with identical measured characteristics from different areas, we would produce a distribution of rate ratios that are always greater or equal to 1. The MRR is the median of this distribution of rate ratios. The smallest possible value of the MRR is 1, which means there is no heterogeneity in the outcome between geographic areas. MMR values greater than 1 indicate heterogeneity in the outcome between geographic areas. The attenuation of the MRR after including individual- and area-level characteristics can identify potential characteristics associated with the variation between geographic areas.

In this paper, we will illustrate the application of the MRR in a multilevel Poisson regression to quantify the magnitude of GCE in SARS-CoV-2 infection using data from Ontario, Canada. Second, we assess how the heterogeneity in SARS-CoV-2 infection between areas measured by the MRR is associated with individual- and area-level characteristics. Finally, we aim to inform investigators about the potential opportunities and challenges in applying the MRR (and similar measures) in future multilevel studies in population health research.

## Methods

### Study design, setting, and population

We conducted a population-based, multilevel observational study using census, laboratory, and health administrative data. The study population included community-dwelling adults in Ontario, Canada. Ontario residents are covered by a universal, publicly funded health care plan. Ontario had substantial geographic variation in SARS-CoV-2 infection rates [6]. The study period included SARS-CoV-2 infections from March 1, 2020, to May 1, 2021. This study period encompasses Ontario’s first to third COVID-19 waves before the general adult population was eligible for COVID-19 vaccination [24]. Our multilevel study included two levels: individuals nested into areas. The area level was the census geographic unit of the forward sortation area (FSA), representing the first three characters of the six-character Canadian postal code [25].

### Data sources, linkages, and inclusion criteria

We captured laboratory-identified SARS-CoV-2 infections using Ontario Laboratories Information System (OLIS) data and linked this information to relevant health administrative and census data. These datasets were linked using unique encoded identifiers and analyzed at ICES (formerly the Institute for Clinical Evaluative Sciences) [26]. The OLIS captured approximately 90% of all laboratory-identified SARS-CoV-2 infections reported in Ontario [6].

We obtained individual-level data from the Registered Persons database, the Ontario Health Insurance Program, the Canadian Institute for Health Information Discharge Abstract Database, the National Ambulatory Care Reporting System, the Continuing Care Reporting System, and the Ontario Drug Benefit claims database. We used validated algorithms to identify chronic disease conditions in the administrative data [27–31]. We obtained area-level information at the FSA using the 2016 Canadian Census data linked using the Postal Code Conversion File (PCCF + 2016, Version 7B) [32]. The 2016 Canadian census area profiles contain 513 FSAs for Ontario, with a median population of 22,260 [33]. The 2016 census FSAs for Ontario were mapped in Additional file 1: Figure S1.

If a person had more than one positive SARS-CoV-2 test during the study period, only the first positive test result was used. The SARS-CoV-2 infection cases included Ontario adults, 20 to 114 years old, with a laboratory-confirmed SARS-CoV-2 infection who were alive at the start of the study period. Individuals were excluded if they were missing age and postal code information, were not eligible for Ontario Health Insurance, or were residing in a long-term care facility in the 90 days before March 1, 2020. The Ontario population used as the offset variable for rates in this study included Ontario adults from the Ontario register data, age 20 to 114 years old, alive at the start of the study period. Individuals were excluded if they were missing postal code information, were not eligible for Ontario Health Insurance, or were residing in a long-term care facility in the 90 days before March 1, 2020.

### Measures

For the study outcome, we investigated test-positive SARS-CoV-2 infection rates per 1000 people during the study period [34]. The study outcome is interpreted as a rate because we included the Ontario population as an offset (or exposure) variable for unequal exposure in the population size at risk [22].

We selected individual-level variables previously shown to be associated with SARS-CoV-2 test positivity in Ontario [6]. The individual-level variables included age (20–34, 35–49, 50–64, 65–114), sex (male, female), history of asthma (yes, no), history of diabetes (yes, no), history of hypertension (yes, no), history of congestive heart failure (CHF) (yes, no), and history of chronic obstructive pulmonary disease (COPD) (yes, no). Several area-level characteristics have established associations with geographic variation in SARS-CoV-2 rates [35–37]. We first created a comprehensive list of potential census variables for study inclusion using eight broad domains: age, ethnicity, family characteristics, immigration, income, labour, language, and education. Because census variables are often strongly correlated [38], we used the SAS VARCLUS procedure to conduct a hierarchical cluster analysis of the census variables to inform FSA census-based variable selection and reduce multicollinearity [39]. The FSA census-based variables included were household size, median after-tax household income, the proportion of employed people in sales/service jobs, and the proportion of people who primarily drive to work. Further details about the definitions of each census variable used in the study are included in Additional file 1: Table S1. To aid comparison between the area-level variables and to further reduce potential multicollinearity in our study, the area-level covariates were standardized to have a mean of zero and a standard deviation of 1.

### Statistical analyses

Before fitting these regression models, we aggregated the person-level data by summing the number of test-positive SARS-CoV-2 infections and the number of people in the Ontario population at risk separately across the different covariate combinations of individual and area-level variables. This meant that each row of the aggregated data represented all the cases and the Ontario population who shared the same individual-level and area-level characteristics. Aggregating the data increases the computational efficiency of the statistical analysis when the data is large. We include a schematic diagram of the aggregated data structure in the Additional file 1: Figure S2. Due to rate instability concerns, we excluded rows with a population size of less than 20 people. We also excluded rows with missing census variables.

Because of the hierarchical structure of study data (with individuals nested within FSAs) and the outcome was a rate (i.e., the rate of test-positive SARS-CoV-2 infection per 1000 people), we applied multilevel Poisson regression with FSA-specific random intercepts [22]. We adopted a sequential modelling strategy [14]. Model I was the null model. We analyzed the quantified variation in the rate of SARS-CoV-2 infection before accounting for any individual or area variables. In model II, we included the seven individual-level variables. We expanded model II by including the four area-level variables in model III. We calculated the proportional change in the FSA variance in models II and III to assess how adding individual- and area-level characteristics accounts for some of the FSA variance in the null model [40] We also evaluated how individual and area-level covariates might account for GCE measured by MRR, by adapting the formula for the percentage excess risk explained to measure the attenuation of the MRR after including each set of variables [41, 42]: (MRR_U_ – MRR_A_) / (MRR_A_ – 1) * 100. The MRR_U_ represents the unadjusted MRR from the null model as the reference, and the MRR_A_ represents the MRR from each subsequent model in the sequential model-building strategy.

We conducted additional analyses to ensure adequate model fit and robustness of the results. We graphically assessed the linearity assumption between the continuous FSA census-based variables and the outcome using restricted cubic splines. Model fit statistics were produced for each model using the deviance, Akaike’s information criterion (AIC), and the Bayesian information criterion (BIC). A key distributional assumption of the Poisson regression model is equidispersion; the response variable’s variance equals the mean [43]. When the Poisson regression model is extradispersed, the variance in the response variable is smaller than the mean (underdispersed), or the variance in the response variable is larger than the mean (overdispersion). Overdispersion is especially concerning because it underestimates the standard errors. All models were assessed for equidispersion by examining whether the dispersion statistic was approximately 1. We also conducted sensitivity analyses to determine the robustness of results to changes in the study period (i.e., COVID-19 waves) and geographic unit of analysis. We used SAS Enterprise Guide v.8.15 (SAS Institute Inc, Cary, NC) for all analyses. The SAS GLIMMIX procedure was used to estimate the multilevel Poisson regression models [44].

## Results

### Descriptive statistics

**Fig. 1:**
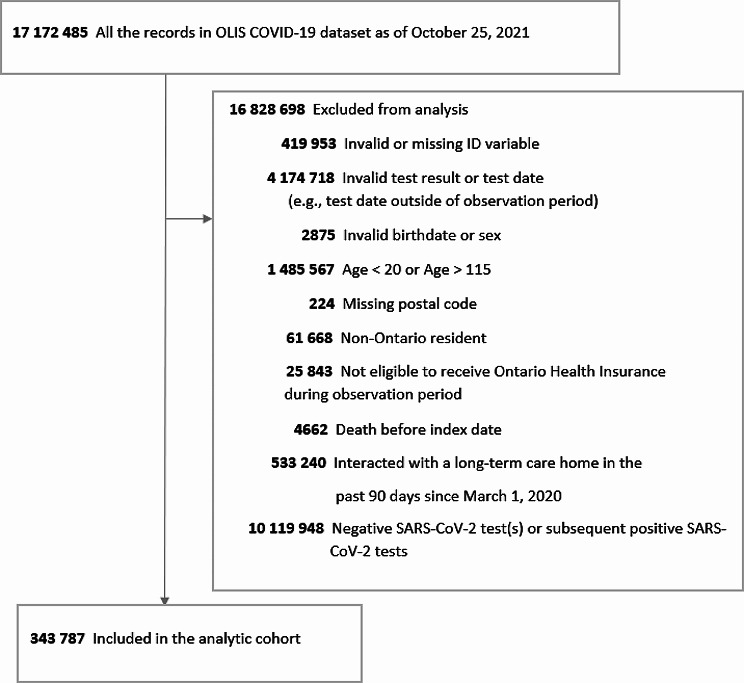
SARS-CoV-2 infection cases flow diagram *Abbreviation* COVID-19: coronavirus disease 2019; OLIS: Ontario Laboratories Information System

The study flowchart for the cases is shown in Fig. 1, and the study flowchart for the Ontario population counts used as the offset is shown in the Additional file 1: Table S2. The cases included a total of 343 787 individuals (median age, 44 years, [interquartile range {IQR}, 30–57; range, 20–107 years]; 51% female) with a SARS-CoV-2 infection between March 1, 2020, and May 1, 2021. Table 1 shows the distribution of the study cohort’s demographic, chronic health conditions, and census-based area-level characteristics. Hypertension (22%) and asthma (15%) were the most prevalent chronic conditions. The Ontario population included 11 789 128 individuals (median age, 49 years, [IQR 34–63; range, 20–114]; 51% female). Except for the oldest age category, the summary distribution of the demographic, chronic conditions, and area-level characteristics in the Ontario population are similar to those who tested positive for SARS-CoV-2. After additional exclusion after data aggregation for rows with population sizes less than 20 and missing FSA census-based variables, the analytic study cohort used in the subsequent regression analyses had 342 779 SARS-CoV-2 cases, and the Ontario population size used as the offset was 11 762 208.

**Table 1:** Baseline characteristics of individuals with a SARS-CoV-2 infection and the Ontario population

	No. (%)	
	Tested positive for SARS-CoV-2 (numerator)	Ontario Population (denominator)
**Demographic**		
Total No.	343 787	11 789 128
**Individual-level variables**		
Age (y), median (IQR) [range]	44 (30–57) [20–107]	49 (34–63) [20–114]
Age, y		
20–34	114 497 (33)	3 039 354 (26)
35–49	95 398 (28)	3 023 852 (26)
50–64	88 343 (26)	3 144 045 (27)
65–114	45 549 (13)	2 581 877 (22)
Sex, **n (%)**		
Female	174 037 (51)	6 014 961 (51)
Male	169 750 (49)	5 774 167 (49)
Chronic conditions, **n (%)**		
Diabetes	45 372 (13)	1 385 101 (12)
Hypertension	75 503 (22)	2 953 037 (25)
COPD	16 905 (5)	861 869 (7)
CHF	5788 (2)	226 328 (2)
Asthma	51 566 (15)	1 704 005 (14)
**FSA census-based variables**		
Average household size (n), median (IQR)	2.7 (2.5–3.1)	2.6 (2.2–3.1)
Proportion of employed people driving to work (%), median (IQR)	0.73 (0.58–0.80)	0.77 (0.63–0.86)
Proportion of employed people in sales/service jobs (%), median (IQR)	0.24 (0.22–0.27)	0.23 (0.19–0.28)
Median Household After-tax Income in 2015 (CAD $), median (IQR)	29 373 (24 944 − 33 186)	31 616 (26 240 − 37 120)

*Abbreviations* COPD, chronic obstructive pulmonary disease; CHF, congestive heart failure; CAD, Canadian; y, year; n, number

Note: More details about the definitions of the FSA census-based characteristics are provided in the Additional file: Table S1

The observation period is between March 1, 2020, to May 1, 2021

### Multilevel regression analyses

Table 2 shows the estimated incidence rate ratios and 95% confidence intervals from the three multilevel Poisson regression models that were sequentially adjusted using demographic characteristics, chronic conditions, and area-level characteristics. In the null multilevel Poisson regression model (model I), the MRR was 2.1 per 1000 people. This means that, on average, the rate of SARS-CoV-2 infection is 110% higher in one FSA compared to another randomly selected FSA. The MRR from the null model represents how much heterogeneity in the outcome is attributed to the difference between clusters before including additional variables.

After adjusting for the individual-level characteristics (i.e., age, sex, and chronic conditions) (model II), the MRR was attenuated to 2.07 per 1000 people (MRR attenuated by 3%). This means that, on average, the rate of SARS-CoV-2 infection is 107% higher between two randomly selected individuals with the same individual-level characteristics from two randomly selected FSA ordered by rates. The small attenuation of the MRR also coincided with a small 4.97% proportional change in the variance of the FSA random effect. This suggests that the individual-level variables in the model accounted for little of the between-FSA heterogeneity – the FSA contextual effect – in the rates of SARS-CoV-2. While adjusting for the other variables (i.e., individual binary or categorical values set to the reference level, and random effect being set to the same FSA) [45], an age gradient was observed from the youngest to oldest age group, where young adults had a higher incidence rate of SARS-CoV-2 infection per 1000 people compared to the oldest adult group. In addition, after adjustment for the other covariates, individuals with diabetes and congestive heart failure had a 20% and 30% higher incidence rate of SARS-CoV-2 infection per 1000 people compared to people who did not have these conditions, respectively.

After adjusting for both individual- and FSA census-based characteristics (model III), the MRR was attenuated to 1.67 per 1000 people (MRR attenuated by 39% from the null MRR). This means that, on average, the rate of SARS-CoV-2 infection is 67% higher between two randomly selected individuals with the same individual-level and FSA census-based characteristics from two randomly selected FSA ordered by rates. The large attenuation of the MRR also coincided with a large 52.46% proportional change in the variance of the FSA random effect. This suggests that individual-level and FSA census-based characteristics in the model accounted for a sizeable portion of the between-FSA heterogeneity – the FSA contextual effect – in the rates of SARS-CoV-2. After adjusting for the individual-level and FSA census-based characteristics, the results suggest that the incidence rate of SARS-CoV-2 infection per 1000 people in an FSA increased by 83% for each 1 unit increase in the standard deviation from the mean household size. In addition, after adjusting for the other covariates, the results suggest that the incidence rate of SARS-CoV-2 infection per 1000 people in an FSA decreased by 32% for each 1 unit increase in the standard deviation from the mean proportion of the labour force that primarily drives to work. After adjusting for both individual- and area-level characteristics, the magnitude of the individual-level rate ratios was not altered by including the area-level characteristics.

Because the MRR is on the ratio scale, it allows for comparing its magnitude with the association between the explanatory variables in the study and the outcome. In model III, the MRR was 1.67 per 1000 people, and the reciprocal of the MRR (i.e., 1/1.67) was 0.60 per 1000 people. In examining the rate ratios (excluding categorical age), 0 of the 6 individual-level characteristics had a rate ratio that exceeded the MRR interval (0.60,1.67), and 1 of the 4 FSA census-based variables had one that lay outside of the MRR interval. Household size had a rate ratio of 1.83 per 1000 (95% CI: 1.71–1.97), which exceeded 1.67. The magnitude of the FSA contextual effect (or clustering in an FSA) was larger than 9 out of 10 binary individual-level and continuous FSA census-based variables included in the study. This indicates that between-FSA variation on SARS-CoV-2 infection rates appears greater than the effect of the explanatory variables in the study.

The null model had a dispersion statistic of 1.27, which suggests the Poisson model was overdispersed. However, this is likely apparent overdispersion due to missing explanatory variables rather than real overdispersion, given that the subsequent models had a dispersion statistic close to 1 [46]. Therefore, the Poisson regression model fits the data well. The results of the graphical assessment of the linearity assumption are included in Additional file 1: Figure S3. Across each continuous FSA census-based variable and the predicted rate of the outcome in the fully adjusted model, the lines are close to linear, except at the 95% tail for the proportion of people driving to work. Therefore, the linearity assumption is reasonable for these variables in our study. The additional model fit statistics – deviance, AIC and BIC– represent how well each model fits the data [47]; lower values indicate better model fit. Model III had the best model fit across all three models. There were large decreases in the model fit statistics between model I to model II, but modest decreases in the fit statistics between model II and model III. Although including the FSA census-based variables did not substantially alter the model fit, it did account for a large proportion of the unexplained variance in the outcome between the FSAs based on the proportional change in the variance and the attenuated MRR. Furthermore, the results were robust regarding changes in the study period and geographic unit of analysis, as shown in Additional file 1: Tables S3-S6.

**Table 2:** Sequential multilevel Poisson regression models for individuals with a SARS-CoV-2 infection in Ontario

Distinct Areas (i.e., FSA) = 513 Individuals (Numerator) = 342 729 Population (Denominator) = 11 762 208	Model I (Null model)	Model II (Model I + age, sex, and chronic conditions)	Model III (Model II + census-based area measures)
Intercept	2.97	2.39	2.86
**Individual Level**	**RR (95% CI)**	**RR (95% CI)**	**RR (95% CI)**
Age, yr			
20–34		2.24 (2.18–2.31)	2.24 (2.18–2.31)
35–49		1.84 (1.79–1.89)	1.84 (1.79–1.89)
50–64		1.65 (1.62–1.68)	1.65 (1.62–1.68)
65–114		1.00	1.00
Sex (ref. male)		1.00 (0.99–1.01)	1.00 (0.99–1.01)
Diabetes		1.29 (1.27–1.32)	1.29 (1.27–1.32)
Hypertension		1.09 (1.08–1.10)	1.09 (1.08–1.10)
COPD		0.94 (0.92–0.96)	0.94 (0.92–0.96)
CHF		1.20 (1.16–1.24)	1.20 (1.16–1.24)
Asthma		1.03 (1.01–1.05)	1.03 (1.01–1.05)
**FSA census-based variables**	**RR (95% CI)**	**RR (95% CI)**	**RR (95% CI)**
Household size			1.83 (1.71–1.97)
Proportion of people driving to work			0.68 (0.65–0.71)
Proportion of people in sales / service Jobs			1.13 (1.06–1.21)
Median after-tax income in 2015			0.96 (0.89–1.02)
**Level 2 (FSA) Variance of Random Effect**	0.61	0.58	0.29
Proportional change in variance (PCV) by the new model (%)	Reference	4.92	52.46
Median Rate Ratio	2.1	2.07	1.67
Median Rate Ratio attenuation (%)	Reference	2.73	39.09
**Model Fit Statistics**			
Deviance (-2 Log Likelihood)	178298.7	159760.8	159427.7
Dispersion (Pearson Chi-Square / DF)	1.27	1.04	1.04
AIC	178302.7	159782.8	159457.7
BIC	178311.1	159829.4	159521.3

*Abbreviation* FSA, forward sortation area; RR, incidence rate ratio; CI, confidence interval; yr, year; ref., reference; COPD, chronic obstructive pulmonary disease; CHF, congestive heart failure; DF, degrees of freedom; AIC, Akaike information criterion; BIC, Bayesian information criterion

*Note* The area was defined according to forward sortation areas (FSA). In Canada, the FSA is a geographic region based on the first three characters of the six character Canadian postal code. The FSA census-based variables were standardized to a mean of 0 and a standard deviation of 1. The patients in this study had a SARS-CoV-2 infection in Ontario, Canada between March 1, 2020, and May 1, 2021. The reported intercepts are not exponentiated. The incidence rate ratio for test positive SARS-CoV-2 infection is per 1000 people

## Discussion

We conducted a multilevel analysis to illustrate the utility of the MRR as a summary measure to quantify the magnitude of the GCE in SARS-CoV-2 infection and whether it could be explained by individual- and area-level characteristics in our study. In the fully adjusted model, the MRR was attenuated by approximately 40% from 2.1 in the null model to 1.67 per 1000. This means the rate of SARS-CoV-2 infection is 67% higher between two randomly selected individuals with the same individual-level and FSA census-based characteristics from two randomly selected FSA ordered by rates. However, a large FSA contextual effect still exists in the rate of SARS-CoV-2 infection even after accounting for the individual-level and FSA census-based variables in the study. The fully adjusted MRR of 1.67 was still larger than 9 out of the ten binary individual-level and continuous FSA census-based variables included in the study. In a prior study examining disparities in COVID-19 mortality, the authors described their attenuated MRR of 1.7 as a “fairly large contextual effect” [48]. This suggests that other factors not included in our study may explain even more of the between-FSA heterogeneity in the rates of SARS-CoV-2 infection. For example, our analysis did not include environmental measures (e.g., ambient air pollution) that have been shown to affect respiratory viral infection rates and may explain some of the unexplained variability between the FSAs [49]. Environmental risks often disproportionately impact socially disadvantaged groups and may be more amendable to intervention than the FSA census-based variables in our study [50].

Our study revealed a strong association between larger household sizes in a FSA and a higher rate of SARS-CoV-2 infection. This finding aligns with several other studies identifying a similar relationship [37, 51]. The higher infection rate is likely caused by close and frequent contact with people indoors. Larger household sizes are often associated with smaller physical house sizes, poor housing conditions (e.g., ventilation), more people working outside the home as essential workers, and more household members sharing a room [51]. Public health investment and policy recommendations to provide essential workers housing options to isolate outside of their homes, investments in housing, and better protective gear for essential workers are potential targets for intervention.

We identified a negative association between an increased number of people driving to work in an FSA and a lower rate of SARS-CoV-2 infection. After driving to work, the second most common form of commuting is public transportation, then walking or cycling. Several studies have identified a relationship between public transportation and risk of SARS-CoV-2 infection [52, 53]. The reduced infection risk in people driving to work is likely associated with avoiding close contact with people that would have occurred on public transportation. The lack of ventilation and crowded public transportation systems can increase the risk of infection on public transportation systems compared to driving. Policy recommendations to support working from home to reduce public transport crowding and improved ventilation systems in public transportation may be potential targets for intervention.

The heterogeneity in the between-FSA rates accounted for by the variables in the study can, through attenuation of the MRR, alert policymakers to factors to address at the population level and more explicitly consider how much between-region heterogeneity would still exist after accounting for individual- and area-level characteristics. This can inform important decisions, such as prioritizing resources and suggesting potentially modifiable intervention targets that may have the greatest impact. For example, out of the explanatory variables included in our study, our findings suggest potential interventions to address social determinants of household size may have the most influence on reducing the between-FSA rates of SARS-CoV-2. The MRR can be used as a summary measure to monitor the heterogeneity of an outcome between regions. For example, it can assess the before-and-after impact of a large-scale policy intervention on addressing the heterogeneity of an outcome between regions.

Our study results need to be interpreted considering the completeness of the individual model. Our study lacks individual- and area-level variables of the same sociodemographic factors (e.g., individual-level median income and average median-level income in the FSA). Previous research has shown that individual– and area-level measures do not measure the same construct [54–57]. The lack of individual-level measures of the same sociodemographic factors as the area-level measures makes it unclear whether the variation explained in the MRR by the area-level variables would disappear after including the corresponding individual-level variables. Therefore, we cannot parse whether the individual or area-level variables explain more FSA-level variation. One of the proposed potential benefits of the MRR is the ability to compare the magnitude of the MRR to the magnitude of the fixed effect rate ratios [22]. Our results suggest that compared to individual- and area-level variables included in our study, unmeasured factors in the FSA may have more relevance to the rate of SARS-CoV-2 infection. However, it is often difficult to compare the magnitude of measures of association given differences in the underlying units of the variable, even when the variable is standardized [41, 58]. Our analyses were focused on the MRR, but future studies can explore the inclusion of the variance partition coefficient to understand the systematic differences between geographies [22].

Some limitations of this study should be noted. The results of our study of positive test results for SARS-CoV-2 were conditioned on being a laboratory-confirmed case from a lab that provided data to OLIS. Our analysis assumes that the distribution of the positive SARS-CoV-2 infections are randomly distributed across the FSAs in Ontario. However, the relationship between the individual- and area-level variables on the between-FSA heterogeneity is likely different in individuals with a positive test result for SARS-CoV-2 not represented in OLIS or had a SARS-CoV-2 infection that was not laboratory confirmed. In addition, geographical differences in access to tests and testing strategies may influence rates in ways unaccounted by the varying FSA random intercepts used in our study. Our analysis used FSA-specific random intercepts that assume the infection incidence rate for individuals with a given set of characteristics varies between FSA, and the association (or slope) between infection incidence rate and explanatory variables is consistent (on average) across all the FSAs [14]. However, the association between the outcomes and the explanatory variables may vary across FSAs. In Ontario, some priority groups (i.e., health care workers) and settings (i.e., high-risk congregate settings) received COVID-19 vaccinations before May 1, 2021. We were not able to account for these individuals in our analysis.

The Canadian census data are only collected every five years, and because 2021 data were not available, we used 2016 census data, which has the potential for misclassification [38]. The 2016 census data might not accurately reflect current geographic areas, especially areas affected by recent gentrification and rapid development. In addition, the 2016 census data cannot capture how people were affected by the COVID-19 pandemic (e.g., job loss) and how people changed their behaviours in response to the pandemic (e.g., drove instead of taking public transport). The use of census data can result in the modifiable areal unit problem because a particular census geography might not reflect the most relevant spatial units [38]. However, in our sensitivity analysis, our results were robust even when we changed the geographic unit from FSA to dissemination area in Additional file 1: Table S6. Our multilevel Poisson regression analysis was non-spatial because the spatial proximity of the FSAs was not directly modelled. A non-spatial multilevel analysis with random effects for geographic clusters can indirectly account for some spatial structure. However, our multilevel model does not allow for spatial smoothing or account for spatial autocorrelation. such as more complex hierarchical Bayesian models [5, 59, 60]. If strong spatial autocorrelation exists, this could bias estimates and underestimate the variance. However, the multilevel Poisson model is better at handling large population-based data and is easier to implement than hierarchical Bayesian models.

The main challenge in implementing multilevel models is the need for multilevel data that contains relevant individual- and area-level variables. The accuracy of the measure of geographic variation and the potential relevance of the individual- and area-level variables is determined by including relevant variables, especially individual-level variables. The lack of relevant individual data is common in multilevel studies [61, 62]. The existing tutorials on summary measures of the magnitude of geographic variation tend to focus more on theory, application, and interpretation [22, 23, 45, 63] rather than bias and study design. In our application of these models, we have highlighted some of the challenges to interpretation, considering study design limitations and strategies for dealing with potential sources of error. Our analysis assumed a steady-state population and a constant incidence rate over the study period [64]. A multilevel survival analysis using the median hazard ratio may be more appropriate for longitudinal studies interested in modelling interactions, competing risks, variable person-time at-risk with changing immunity status, or variable incidence rate over time. Future applications of the MRR would be improved by examples of how to compute credible intervals for the MRR using commonly available software (e.g., SAS, R, and Stata). Future multilevel studies should continue to consider the theoretical underpinnings and strategies to overcome potential threats of validity when applying these methods [12–14, 65].

## Conclusions

Understanding how social determinants affect population health outcomes and measuring how between-region heterogeneity in health outcomes are associated with individual- and area-level characteristics is an important goal in population health research. The use of multilevel models with the inclusion of summary measures of area-level variation (i.e., MRR, MOR, MHR) could help move closer to this goal. This study has demonstrated how MRR and similar measures could be valuable to the population health toolkit to measure geographic inequities in population health outcomes and understand potential factors driving the heterogeneity.

## Electronic supplementary material

Below is the link to the electronic supplementary material.

Supplementary Material 1: Additional file 1: Figure S1, Ontario FS, Area (FSA), Map, Table S. Definitions of census-based area characteristics: constructs, statistical units, and operational definitions using the Ontario, Canada, census area profiles, 2016. Figure S2. Schematic diagram of the aggregated data structure used with Poisson multilevel models. Table S2. Ontario population study flow table. Figure S3. Graphical assessment of the linearity assumption of the FSA census-based continuous variables and the rate of SARS-CoV-2 infection using restricted cubic splines in the fully adjusted multilevel Poisson regression. Figure S4. Raw and Pearson Residuals vs Predicted Values Plots. Table S3. COVID-19 Wave 1 Analysis: Sequential multilevel Poisson count regression models for individuals with a SARS-CoV-2 infection in Ontario, Canada between March 1, 2020, and July 31, 2020. Table S4. COVID-19 Wave 2 Analysis: Sequential multilevel Poisson count regression models for individuals with a SARS-CoV-2 infection in Ontario, Canada, between August 1, 2020, and March 1, 2021. Table S5. COVID-19 Wave 3 Analysis: Sequential multilevel Poisson count regression models for individuals with a SARS-CoV-2 infection in Ontario, Canada, between March 2, 2021, and May 1, 2021. Table S6. Dissemination Area Sensitivity Analysis: Sequential multilevel Poisson count regression models for individuals with a SARS-CoV-2 infection in Ontario, Canada, between March 1, 2020, and May 1, 2021 (PDF 619 kb).

## Data Availability

The dataset used in this study is held securely in coded format at ICES. ICES is a prescribed entity under section 45 of Ontario’s Personal Health Information Protection Act. Section 45 authorizes ICES to collect personal health information, without consent, for the purpose of analysis or compiling statistical information with respect to the management of, evaluation or monitoring of, the allocation of resources to or planning for all or part of the health system. Legal restrictions and data sharing agreements prohibit ICES from making the dataset publicly available. Access may be granted to those who meet the conditions for confidential access, available at https://www.ices.on.ca/DAS

## Abbreviations

AIC: Akaike information criterion
BIC: Bayesian information criterion
CHF: Congestive heart failure
COPD: Chronic obstructive pulmonary disease
COVID-19: CORONAVIRUS disease 2019
FSA: Forward sortation area
GCE: General contextual effect
IQR: Interquartile range
MHR: Median hazard ratio
MOR: Median odds ratio
MRR: Median rate ratio
OLIS: Ontario Laboratories Information System database
PCCF: Postal code conversion file
SARS-CoV-2: Severe acute respiratory syndrome coronavirus 2
