# Supplementary material for: Quantifying the magnitude of the general contextual effect in a multilevel study of SARS-CoV-2 infection in Ontario, Canada: application of the median rate ratio in population health research
Source: Popul Health Metr. 2024 Oct 7;22:27. doi: 10.1186/s12963-024-00348-8 (PMC11457329; doi:10.1186/s12963-024-00348-8)
Supplement: Supplementary file 1 — Supplementary Material 1: Additional file 1: Figure S1, Ontario FS, Area (FSA), Map, Table S. Definitions of census-based area characteristics: constructs, statistical units, and operational definitions using the Ontario, Canada, census area profiles, 2016. Figure S2. Schematic diagram of the aggregated data structure used with Poisson multilevel models. Table S2. Ontario population study flow table. Figure S3. Graphical assessment of the linearity assumption of the FSA census-based continuous variables and the rate of SARS-CoV-2 infection using restricted cubic splines in the fully adjusted multilevel Poisson regression. Figure S4. Raw and Pearson Residuals vs Predicted Values Plots. Table S3. COVID-19 Wave 1 Analysis: Sequential multilevel Poisson count regression models for individuals with a SARS-CoV-2 infection in Ontario, Canada between March 1, 2020, and July 31, 2020. Table S4. COVID-19 Wave 2 Analysis: Sequential multilevel Poisson count regression models for individuals with a SARS-CoV-2 infection in Ontario, Canada, between August 1, 2020, and March 1, 2021. Table S5. COVID-19 Wave 3 Analysis: Sequential multilevel Poisson count regression models for individuals with a SARS-CoV-2 infection in Ontario, Canada, between March 2, 2021, and May 1, 2021. Table S6. Dissemination Area Sensitivity Analysis: Sequential multilevel Poisson count regression models for individuals with a SARS-CoV-2 infection in Ontario, Canada, between March 1, 2020, and May 1, 2021 (PDF 619 kb). [file 12963_2024_348_MOESM1_ESM.pdf]

Additional file 1

Figure S1. Ontario, Forward Sortation Area (FSA) Map, 2016

Table S1. Definitions of census-based area characteristics**:** constructs**,** statistical units, and operational definitions using the Ontario, Canada, census area profiles, 2016

Figure S2. Schematic diagram of the aggregated data structure used with Poisson multilevel models.

Table S2. Ontario population study flow table

Figure S3. Graphical assessment of the linearity assumption of the FSA census-based continuous variables and the rate of SARS-CoV-2 infection using restricted cubic splines in the fully adjusted multilevel Poisson regression

Figure S4. Raw and Pearson Residuals vs Predicted Values Plots

Table S3. COVID-19 Wave 1 Analysis: Sequential multilevel Poisson count regression models for individuals with a SARS-CoV-2 infection in Ontario, Canada between March 1, 2020, and July 31, 2020

Table S4. COVID-19 Wave 2 Analysis: Sequential multilevel Poisson count regression models for individuals with a SARS-CoV-2 infection in Ontario, Canada, between August 1, 2020, and March 1, 2021.

Table S5. COVID-19 Wave 3 Analysis: Sequential multilevel Poisson count regression models for individuals with a SARS-CoV-2 infection in Ontario, Canada, between March 2, 2021, and May 1, 2021

Table S6. Dissemination Area Sensitivity Analysis: Sequential multilevel Poisson count regression models for individuals with a SARS-CoV-2 infection in Ontario, Canada, between March 1, 2020, and May 1, 2021

Figure S1. Ontario, Forward Sortation Area (FSA) Map, 2016

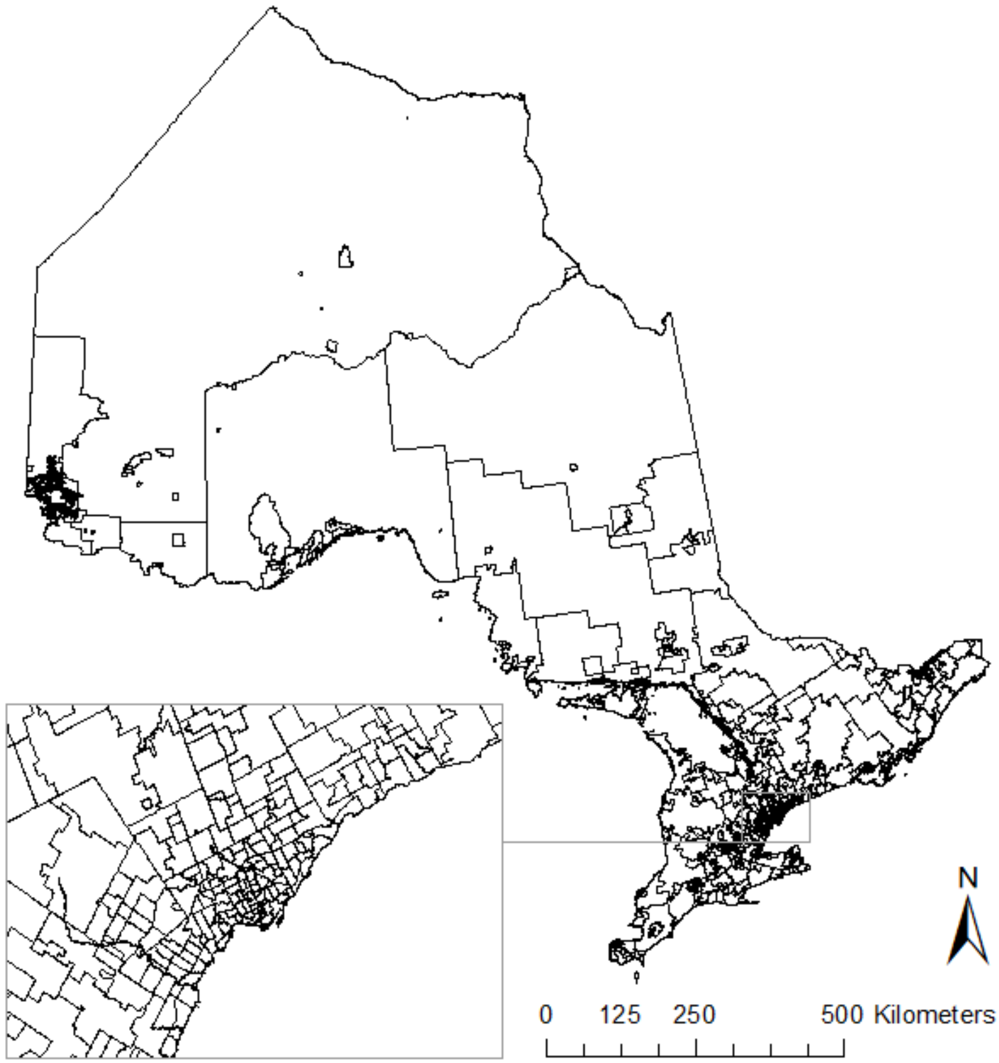

This map shows the forward sortation areas (FSA) in Ontario in 2016. FSAs are regions defined by the first three characters of the six-character Canadian postal code. A thin black line outlines the geographic administrative boundaries of the FSA regions.

Table S1. Definitions of census-based area characteristics: constructs, statistical units, and operational definitions using the Ontario, Canada, census area profiles, 2016

**Construct**	**Statistical Unit**	**Operational Definition (Unit)**	**Census Variables used in the Ontario Census Area Profile (dataset)**
**(A) Occupation Type**			
Proportion of employed people that work in sales and service jobs	Persons	Percentage of total labour force population, aged 15 and older, in private households, which work in sales and service occupations, based on the National Occupational Classification 2016	F67 / F60 (Labour)
**(B) Transportation**			
Proportion of employed people that drive to work	Persons	Percentage of total labour force population, aged 15 and older, in private households, which drives a car, truck, or van as the main mode of transportation between home and work	F197 / F196 (Labour)
**(C) Housing**			
Household size	Private Household	Average number of persons per private household	F18 (Family)
**(D) Income**			
Median after-tax income in 2015	Persons	Median after-tax income in 2015 for the population, aged 15 and older, in private households	F5 (Income)

Note: Private households exclude people living outside of Canada and in collective dwellings (e.g., hospitals, nursing homes, prisons, student residences).

Figure S2. Schematic diagram of the aggregated data structure used with Poisson multilevel models.

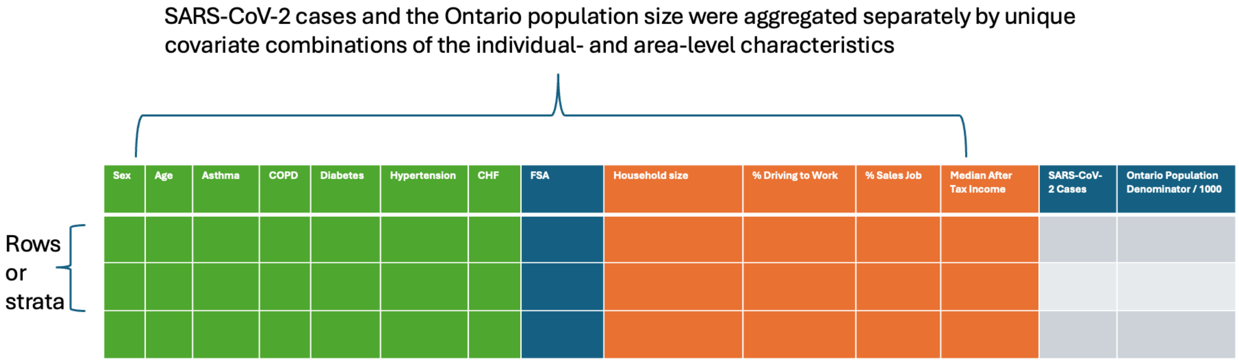

Table S2. Ontario population study flow table

Step	Description	# Excluded	Total Cohort remaining
1	All people in Ontario register data (RPDB) as of 10NOV2021	Before Exclusion	20,837,955
2	Include-Subject can be linked to health admin data and has sex and birth date value in RPDB	0	20,837,955
3	Include-Subject birth year > 1900	39,225	20,798,730
4	Include-Alive on MAR 1 2020	2,771,000	18,027,730
5	Include - 20 <= age <= 114	3,415,433	14,612,297
6	Exclude - No postal code available in the RPDB dataset	252,440	14,359,857
7	Exclude - Non-Ontario resident in the RPDB population control dataset	628,484	13,731,373
8	Exclude - Not eligible to receive Ontario Health Insurance Plan (OHIP) as of the start of observation period	1,845,521	11,885,852
9	Exclude - People who interacted with long-term care home in the past 90 days since Mar 1 2020	96,724	11,789,128
10	Final Ontario population cohort	0	**11,789,128**

Figure S3. Graphical assessment of the linearity assumption of the FSA census-based continuous variables and the rate of SARS-CoV-2 infection using restricted cubic splines in the fully adjusted Poisson regression generalized estimating equation model

A) Household Size

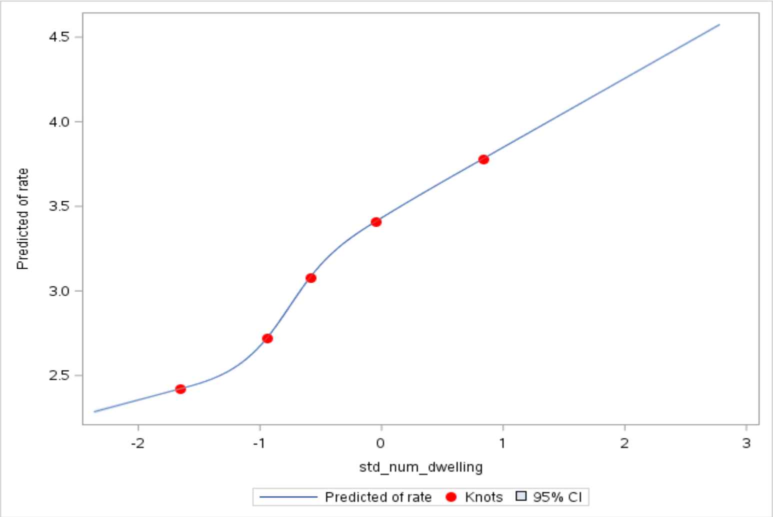

B) Proportion of employed people that work in sales and service jobs

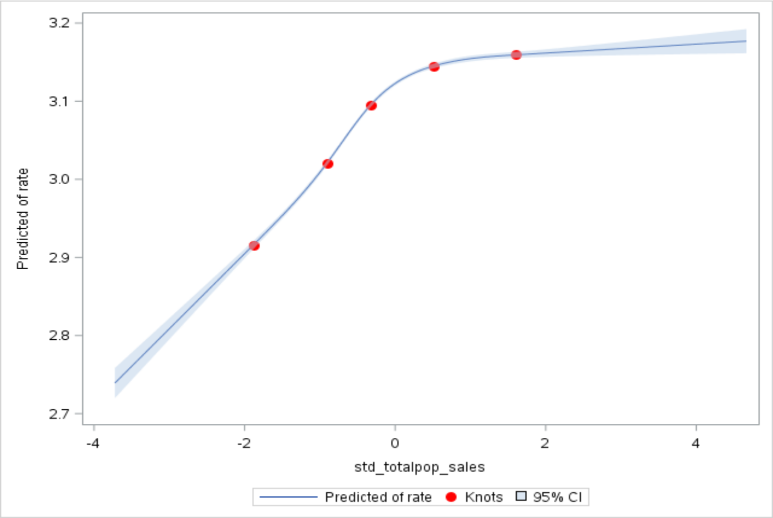

C) Median after-tax income in 2015

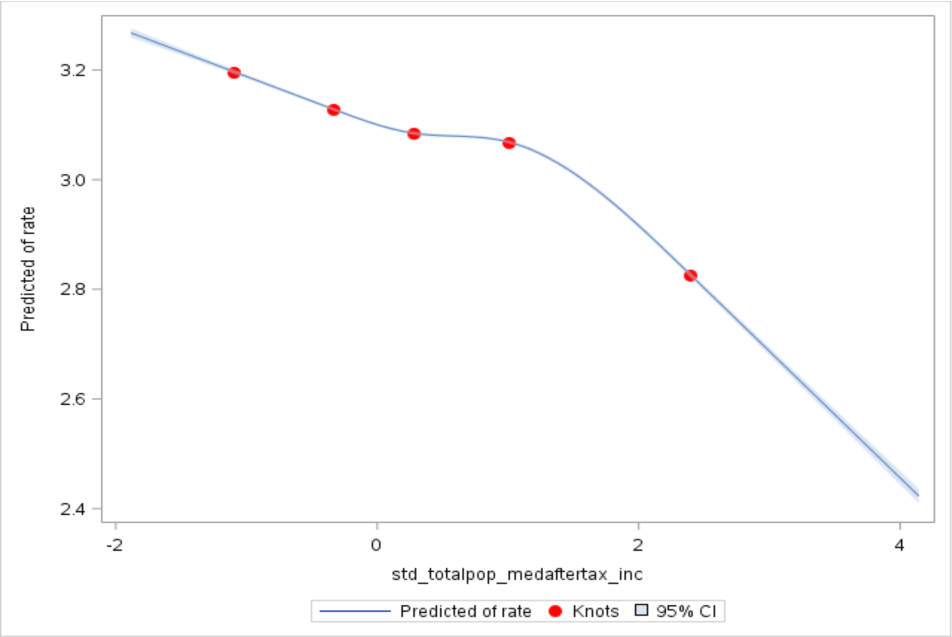

D) Proportion of employed people that drive to work

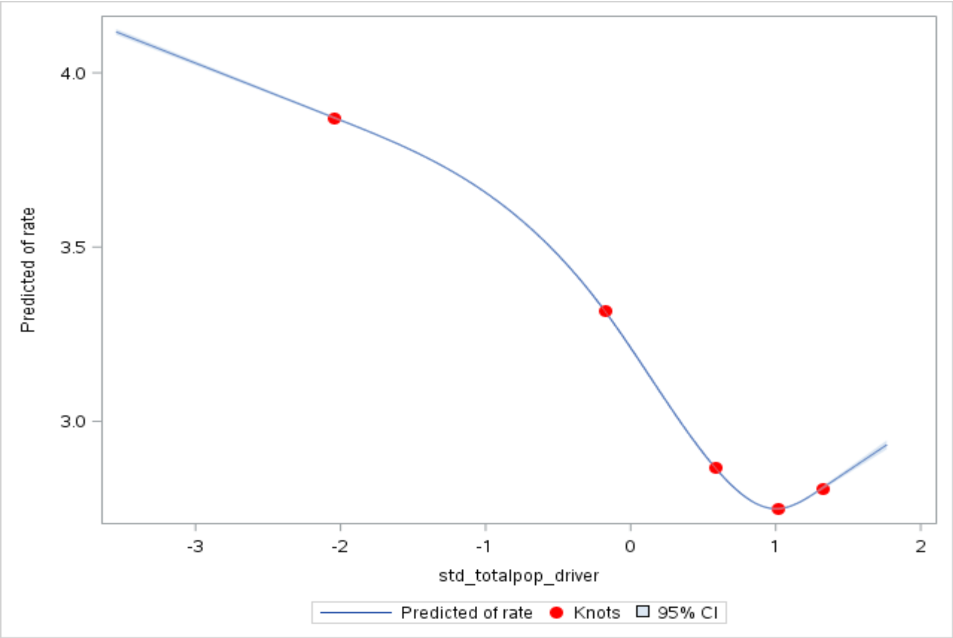

Figure S4. Raw and Pearson Residuals vs Predicted Values Plots

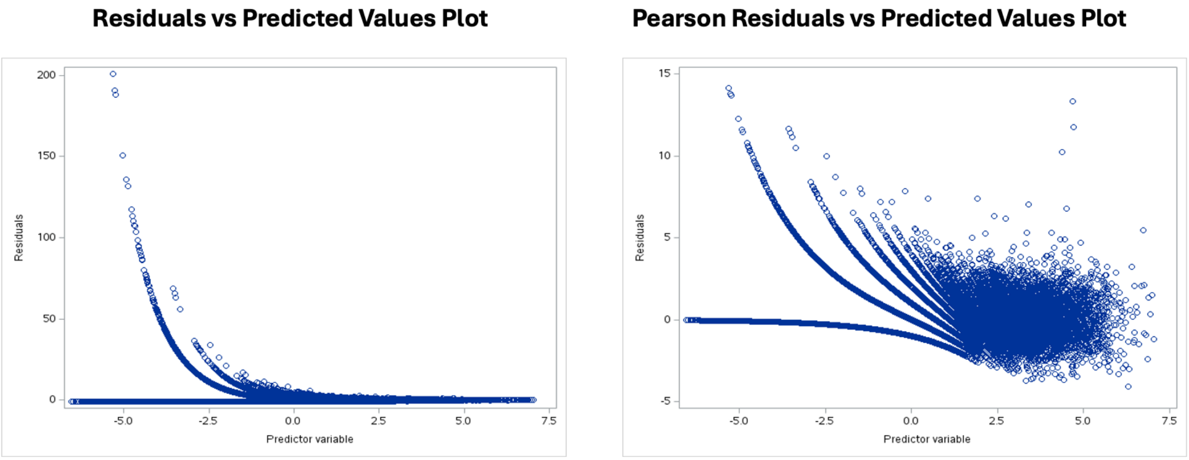

Table S3. **COVID-19 Wave 1 Analysis: Sequential multilevel Poisson count regression models for individuals with a SARS-CoV-2 infection in Ontario, Canada between March 1, 2020, and July 31, 2020.**

	**Model 1 (Null model)**	**Model 2 (Model 1 + age, sex, and chronic conditions)**	**Model 3 (Model 2 + census-based area measures)**
Intercept	0.3367	-0.2431	0.2098
**Individual Level**	**RR (95% CI)**	**RR (95% CI)**	**RR (95% CI)**
Age, yr			
20-34		1.79 (1.63-1.97)	1.79 (1.62-1.96)
35-49		1.64 (1.50-1.79)	1.64 (1.50-1.79)
50-64		1.67 (1.55-1.79)	1.66 (1.55-1.79)
65-114		1.00	1.00
Sex (ref. Male)		1.09 (1.03-1.16)	1.09 (1.03-1.16)
Diabetes		1.35 (1.29-1.42)	1.35 (1.29-1.41)
Hypertension		1.29 (1.23-1.35)	1.29 (1.23-1.35)
COPD		0.98 (0.92-1.04)	0.98 (0.92-1.05)
CHF		1.55 (1.42-1.70)	1.55 (1.42-1.70)
Asthma		0.98 (0.92-1.03)	0.98 (0.92-1.03)
**FSA census-based variables**	**RR (95% CI)**	**RR (95% CI)**	**RR (95% CI)**
Household size			1.80 (1.65-1.96)
Proportion of people driving to work			0.65 (0.61-0.69)
Proportion of people in sales / service Jobs			1.09 (1.01-1.19)
Median after-tax income in 2015			0.95 (0.88-1.03)
**Level 2 (FSA) Variance of Random Effect**	0.71	0.70	0.39
Proportional change in variance (PCV) by the new model (%)	Reference	1.4	45.1
Median Rate Ratio	2.24	2.22	1.82
**Model Fit Statistics**			
Deviance (-2 Log Likelihood)	55667.47	54531.42	54269.79
Dispersion (Pearson Chi-Square / DF)	1.25	1.03	1.03
AIC	55671.47	54553.42	54299.79
BIC	55679.95	54600.06	54363.39

Abbreviations: FSA, forward sortation area; RR, rate ratio; CI, confidence interval; yr, year; ref., reference; COPD, chronic obstructive pulmonary disease; CHF, congestive heart failure; DF, degrees of freedom; AIC, Akaike information criterion; BIC, Bayesian information criterion.

The reported intercepts are not exponentiated. The incidence rate ratio for SARS-CoV-2 infection is per 1000 people.

Table S4. **COVID-19 Wave 2 Analysis: Sequential multilevel Poisson count regression models for individuals with a SARS-CoV-2 infection in Ontario, Canada, between August 1, 2020, and March 1, 2021.**

	**Model 1 (Null model)**	**Model 2 (Model 1 + age, sex, and chronic conditions)**	**Model 3 (Model 2 + census-based area measures)**
Intercept	2.34	1.77	2.27
**Individual Level**	**RR (95% CI)**	**RR (95% CI)**	**RR (95% CI)**
Age, yr			
20-34		2.23 (2.16-2.31)	2.23 (2.16-2.31)
35-49		1.77 (1.72-1.83)	1.77 (1.72-1.83)
50-64		1.62 (1.58-1.66)	1.62 (1.58-1.66)
65-114		Reference	Reference
Sex (ref. Male)		1.01 (1.00-1.02)	1.01 (1.00-1.02)
Diabetes		1.31 (1.29-1.34)	1.31 (1.29-1.34)
Hypertension		1.10 (1.09-1.12)	1.10 (1.09-1.12)
COPD		0.95 (0.92-0.97)	0.95 (0.92-0.97)
CHF		1.25 (1.20-1.30)	1.25 (1.20-1.30)
Asthma		1.01 (0.99-1.03)	1.01 (0.99-1.03)
**FSA census-based variables**	**RR (95% CI)**	**RR (95% CI)**	**RR (95% CI)**
Household size			1.93 (1.79-2.08)
Proportion of people driving to work			0.69 (0.65-0.72)
Proportion of people in sales / service Jobs			1.10 (1.03-1.19)
Median after-tax income in 2015			0.91 (0.84-0.98)
**Level 2 (FSA) Variance of Random Effect**	0.73	0.70	0.37
Proportional change in variance (PCV) by the new model (%)	Reference	4.1	49.3
Median Rate Ratio	2.25	2.22	1.79
**Model Fit Statistics**			
Deviance (-2 Log Likelihood)	134885.5	124780.5	124480.4
Dispersion (Pearson Chi-Square / DF)	1.12	0.98	0.98
AIC	55671.47	54553.42	54299.79
BIC	55679.95	54600.06	54363.39

Abbreviations: FSA, forward sortation area; RR, rate ratio; CI, confidence interval; yr, year; ref., reference; COPD, chronic obstructive pulmonary disease; CHF, congestive heart failure; DF, degrees of freedom; AIC, Akaike information criterion; BIC, Bayesian information criterion.

The reported intercepts are not exponentiated. The incidence rate ratio for a SARS-CoV-2 infection is per 1000 people.

Table S5. **COVID-19 Wave 3 Analysis: Sequential multilevel Poisson count regression models for individuals with a SARS-CoV-2 infection in Ontario, Canada, between March 2, 2021, and May 1, 2021**

	Model 1 (Null model)	Model 2 (Model 1 + age, sex, and chronic conditions)	Model 3 (Model 2 + census-based area measures)
Intercept	1.97	1.39	1.83
**Individual Level**	**RR (95% CI)**	**RR (95% CI)**	**RR (95% CI)**
Age, yr			
20-34		2.30 (2.22-2.39)	2.30 (2.21-2.39)
35-49		1.95 (1.88-2.02)	1.95 (1.88-2.01)
50-64		1.65 (1.61-1.70)	1.65 (1.61-1.70)
65-114		Reference	Reference
Sex (ref. Male)		0.96 (0.95-0.97)	0.96 (0.95-0.97)
Diabetes		1.26 (1.23-1.29)	1.26 (1.23-1.29)
Hypertension		1.03 (1.01-1.05)	1.03 (1.01-1.05)
COPD		0.95 (0.92-0.98)	0.95 (0.92-0.98)
CHF		1.13 (1.07-1.20)	1.13 (1.07-1.20)
Asthma		1.07 (1.05-1.09)	1.07 (1.05-1.09)
**FSA census-based variables**	**RR (95% CI)**	**RR (95% CI)**	**RR (95% CI)**
Household size			1.78 (1.66-1.92)
Proportion of people driving to work			0.66 (0.63-0.69)
Proportion of people in sales / service Jobs			1.20 (1.12-1.28)
Median after-tax income in 2015			1.03 (0.96-1.10)
**Level 2 (FSA) Variance of Random Effect**	0.64	0.61	0.30
Proportional change in variance by the new model (%)	Reference	4.7	53.13
Median Rate Ratio	2.14	2.11	1.69
**Model Fit Statistics**			
Deviance (-2 Log Likelihood)	110312.8	102381.6	102061.6
Dispersion (Pearson Chi-Square / DF)	1.05	0.98	0.98
AIC	110316.8	102403.6	102091.6
BIC	110325.3	102450.2	102155.2

Abbreviations: FSA, forward sortation area; RR, rate ratio; CI, confidence interval; yr, year; ref., reference; COPD, chronic obstructive pulmonary disease; CHF, congestive heart failure; DF, degrees of freedom; AIC, Akaike information criterion; BIC, Bayesian information criterion.

The reported intercepts are not exponentiated. The incidence rate ratio for SARS-CoV-2 infection is per 1000 people.

Table S6. Dissemination Area Sensitivity Analysis: Sequential multilevel Poisson count regression models for individuals with a SARS-CoV-2 infection in Ontario, Canada, between March 1, 2020, and May 1, 2021.

	**Model 1 (Null model)**	**Model 2 (Model 1 + age, sex, and chronic conditions)**	**Model 3 (Model 2 + census-based area measures)**
Intercept	2.98	2.45	2.77
**Individual Level**	**RR (95% CI)**	**RR (95% CI)**	**RR (95% CI)**
Age, yr			
20-34		2.16 (2.12-2.20)	2.14 (2.10-2.18)
35-49		1.78 (1.75-1.81)	1.76 (1.73-1.79)
50-64		1.63 (1.60-1.65)	1.62 (1.59-1.64)
65-114		Reference	Reference
Sex (ref. Male)		1.00 (0.99-1.00)	1.00 (0.99-1.00)
Diabetes		1.23 (1.22-1.25)	1.22 (1.21-1.24)
Hypertension		1.08 (1.07-1.09)	1.08 (1.07-1.09)
COPD		0.87 (0.85-0.88)	0.87 (0.86-0.89)
CHF		1.06 (1.03-1.09)	1.06 (1.03-1.10)
Asthma		1.01 (1.00-1.02)	1.01 (1.00-1.02)
**DA census-based variables**	**RR (95% CI)**	**RR (95% CI)**	**RR (95% CI)**
Household size			1.59 (1.57-1.61)
Proportion of people driving to work			0.77 (0.76-0.78)
Proportion of people in sales / service Jobs			1.03 (1.02-1.04)
Median after-tax income in 2015			0.85 (0.84-0.86)
**Level 2 (DA) Variance of Random Effect**	0.59	0.56	0.32
Proportional change in variance by the new model (%)	Reference	5.4	45.8
Median Rate Ratio	2.07	2.04	1.72
**Model Fit Statistics**			
Deviance (-2 Log Likelihood)	950103.8	933242.9	925758
Dispersion (Pearson Chi-Square / DF)	0.84	0.91	0.91
AIC	950107.8	935318.9	925788
BIC	950123.5	935358.1	925905.5

Abbreviations: DA, dissemination area; RR, rate ratio; CI, confidence interval; yr, year; ref., reference; COPD, chronic obstructive pulmonary disease; CHF, congestive heart failure; DF, degrees of freedom; AIC, Akaike information criterion; BIC, Bayesian information criterion.

Note: The geographic region was defined according to dissemination area (DA). The reported intercepts are not exponentiated. The incidence rate ratio for SARS-CoV-2 infection is per 1000 people.
